# B-cell clonogenic activity of HIV-1 p17 variants is driven by PAR1-mediated EGF transactivation

**DOI:** 10.1038/s41417-020-00246-9

**Published:** 2020-10-22

**Authors:** Cinzia Giagulli, Francesca Caccuri, Simone Zorzan, Antonella Bugatti, Alberto Zani, Federica Filippini, Ekta Manocha, Pasqualina D’Ursi, Alessandro Orro, Riccardo Dolcetti, Arnaldo Caruso

**Affiliations:** 1 Section of Microbiology, Department of Molecular and Translational Medicine, University of Brescia, 25123 Brescia, Italy; 2 Plantech, Environmental Research and Innovation (ERIN) Department, Luxembourg Institute of Science & Technology (LIST), L-4422 Belvaux, Luxembourg; 3 Department of Biomedical Sciences, Institute for Biomedical Technologies e National Research Council (ITB-CNR), 20090 Segrate (MI), Italy; 4 University of Queensland Diamantina Institute, Translational Research Institute, University of Queensland, Brisbane, QLD Australia; 5 Cancer Bio-Immunotherapy Unit, Centro di Riferimento Oncologico – IRCCS, Aviano, Italy

**Keywords:** Cancer microenvironment, Cell biology, Drug development

## Abstract

Combined antiretroviral therapy (cART) for HIV-1 dramatically slows disease progression among HIV^+^ individuals. Currently, lymphoma represents the main cause of death among HIV-1-infected patients. Detection of p17 variants (vp17s) endowed with B-cell clonogenic activity in HIV-1-seropositive patients with lymphoma suggests their possible role in lymphomagenesis. Here, we demonstrate that the clonogenic activity of vp17s is mediated by their binding to PAR1 and to PAR1-mediated EGFR transactivation through Gq protein. The entire vp17s-triggered clonogenic process is MMPs dependent. Moreover, phosphoproteomic and bioinformatic analysis highlighted the crucial role of EGFR/PI3K/Akt pathway in modulating several molecules promoting cancer progression, including RAC1, ABL1, p53, CDK1, NPM, Rb, PTP-1B, and STAT1. Finally, we show that a peptide (F1) corresponding to the vp17s functional epitope is sufficient to trigger the PAR1/EGFR/PI3K/Akt pathway and bind PAR1. Our findings suggest novel potential therapeutic targets to counteract vp17-driven lymphomagenesis in HIV^+^ patients.

## Introduction

Combined antiretroviral therapy (cART) has dramatically slowed HIV-1 disease progression in seropositive individuals, improving their survival and contributing to a decline of AIDS-related mortality [1]. Despite cART, malignant lymphoma still represents the most frequent malignancy and the major cause of mortality among HIV-1-infected patients [1]. The risk of non‐Hodgkin lymphoma (NHL) is increased 60–200 fold in HIV^+^ patients when compared with the general population [2]. Moreover, most HIV-1-related NHLs are high-grade B-cell lymphomas, such as diffuse large B-cell lymphoma (DLBCL) and Burkitt lymphoma (BL), and are characterized by clinical aggressiveness [1]. These lymphomas frequently present with advanced stage involving extranodal sites and have a worse clinical outcome as compared to similar aggressive lymphomas in the general population.

Different mechanisms have been hypothesized for B-cell transformation in HIV^+^ patients, including the pathogenic involvement of HIV-1 structural and regulatory proteins, such as gp120 and Tat, which induce chronic inflammation and B-cell activation and proliferation [3, 4]. More recently, the structural matrix protein p17, which is known to play an essential role in viral assembly and maturation, has been proposed to exert a role in HIV-1-associated lymphomagenesis. P17 protein released by infected cells along the entire virus life cycle was shown to accumulate within lymphoid tissues [5], where it may deregulate the functional activities of various immune cells, including B lymphocytes [6]. In addition, HIV-1 transcription can be efficiently induced by different stimuli, even in the presence of protease inhibitors [7], strengthening the evidence that p17 can be synthesized and released, even under cART, in absence of viral replication [5]. Moreover, the ability of p17 to promote receptor-mediated angiogenesis and lymphangiogenesis of vascular and lymphatic endothelial cells in vitro and in vivo [8, 9] may also contribute to enhance and sustain B-cell proliferation and survival. The importance of angiogenesis and lymphangiogenesis in lymphoma development is now well recognized, as also supported by the observation that alterations of these processes correlated with highly aggressive NHLs [10, 11]. Finally, recent findings have strongly indicated that p17 variants (vp17s) enriched in NHL patients may directly promote B-cell clonogenicity [12–14]. Indeed, several studies highlighted that mutations in the p17 backbone may significantly impact on B-cell signaling pathways known to contribute to B-cell lymphomagenesis [12, 13, 15, 16]. In particular, unlike the wild-type protein (refp17; derived from HIV BH10, Clade B), vp17s isolated from blood and lymphoma tissues of NHL patients were shown to directly stimulate B-cell proliferation by activating the oncogenic PI3K/Akt signaling pathway [12]. These NHL-derived vp17s are characterized by amino acid (aa) insertions in the C-terminus at position 117–118 (Ala–Ala) or 125–126 (Gly–Asn) and by other mutations throughout the sequence. Ultra-deep pyrosequencing showed that these two categories of vp17s are more frequently detected in plasma of HIV^+^ patients with NHL than those not showing evidence of lymphoma [12].

The molecular mechanisms underlying the diverse activity of vp17s have been related to destabilized or altered p17 conformations [12], with the exposure of a functional epitope corresponding to aa 1–20 in the N-terminal region of the protein [14]. Since this epitope doesn’t possess the capability to bind the known p17 receptors [14], CXCR1 and CXCR2, it has been hypothesized that an alternate receptor could also mediate the B-cell growth-promoting effects of vp17. Identification of additional cellular receptors for vp17 is critical to better understand their B-cell clonogenic potential and design mechanism-based strategies of treatment for HIV-associated NHL.

In this study, we provide evidence supporting the role of a new receptor for vp17s together with insights on the molecular mechanisms underlying vp17s-triggered B-cell proliferation. To this aim, we investigated the biologic effects of the refp17 and two clonogenic vp17s isolated from lymphoma patients, NHL-a101 and NHL-a102, which belong to two different categories of vp17s presenting aa insertions at position 117–118 and 125–126, respectively [12]. Our data highlight the role of protease-activated receptor 1 (PAR1) in promoting B-cell clonogenic activity of vp17s through epidermal growth factor receptor (EGFR) transactivation. These findings offer new opportunities to develop new therapeutic interventions towards HIV-1-related lymphoma, and, potentially, also other aggressive cancers occurring in HIV^+^ individuals.

## Materials and methods

### Cell cultures

Human lymphoma B-cell lines Raji and BJAB were obtained from American Type Culture Collection (ATCC, Manassas, Virginia, USA) and cultured in complete medium containing RPMI-1640 medium supplemented with 10% (vol/vol) fetal bovine serum (FBS), 1 mM L-glutamine, 1 mM sodium pyruvate at 37 °C in 5% CO_2_ humidified atmosphere.

### Recombinant proteins

Purified endotoxin (LPS)-free recombinant HIV-1 matrix protein p17s were produced as previously described [12]. Briefly, the coding sequence of HIV-1 isolate BH10 p17 (refp17; amino acids 1–132) was amplified by PCR with specific primers, which allowed us to clone the refp17 sequence into the BamH1 site of the prokaryotic expression vector pGEX-2T (GE Healthcare, Chicago, IL, USA). The vp17s NHL-a101 and NHL-a102 obtained from the lymphoma biopsy of patient a101 and a102, respectively, were amplified by PCR and cloned into BamH1 and XhoI sites of the same vector. The recombinant proteins were further purified (>98%) by reverse-phase fast performance liquid chromatography. The absence of LPS contamination (<0.25 endotoxin units per mL) in protein preparation was assessed by Limulus amoebocyte assay (Associates of Cape Cod, East Falmouth, MA, USA).

### High throughput phosphoproteomic and bioinformatic analysis

Phosphoproteomic and bioinformatic analysis were performed as previously described [16]. Raji cells stimulated or not for 5 min with refp17, NHL-a101 and NHL-a102 (0.1 μg/ml) were lysed and prepared for KinexTM Antibody Microarray (KAM-850), according to manufacturer’s instructions (Kinexus Bioinformatics Corporation, Vancouver, Canada). Cell lysates were checked for pAkt levels by western blot analysis, since p17 proteins are known to modulate PI3K/Akt signaling pathway [12], and then sent to Kinexus Bioinformatics Corporation for hybridization. The array contains 517 pan-specific and 337 phospho-specific probes selected by the company to recognize epitopes on 466 proteins. The raw data, processed by Kinexus with the commercial software ImaGene, were filtered according to criteria equal or stricter than the company recommendations. Only probes matching these criteria where considered for further analysis: signal to noise ratio >1.6 or signal to noise ratio <1.6 and noise inferior than the average noise of the array; maximum variation coefficient <0.2. The analysis was led with custom R, Visual Basic and MySQL procedures. The software is proprietary, with no public license, and available upon request. Z-scores for normalization and z-ratios for comparisons were calculated as previously described [16, 17] and a significative threshold of 1.5 was adopted for z-ratios (manufacturer recommends a threshold of at least 1.1). Since the signal of a single array is noisy and the results should be considered only as indicative, a subsequent multi-immunoblot verification step to rule out false positive array signals was performed according to the manufacturer’s instructions. The 72 most significant probes were selected for a pre-screening step to select the reactive antibodies. Pre-screening allowed to select 36 antibodies and a final multi-immunoblot experiment was led by Kinexus with the reactive antibodies on an aliquot of the same samples used for the Microarray. The multi-immunoblotting was then analyzed by densitometric analysis from Kinexus Bioinformatic Corporation and blot quantification data of vp17 treated samples were compared to refp17 ones for the interpretation of the results. STRING database [18] was used to identify known, experimentally verified interactions as a base for the design of the signal transduction pathways, with a combined score >0.7. Literature data mining was also performed by multiple database interrogations, mainly based on PubMed (http://www.ncbi.nlm.nih.gov/pubmed), PhosphoSite (http://www.phosphosite.org/), Uniprot (http://www.uniprot.org) and Genecards (http://www.genecards.org).

### Human EGFR phosphorylation antibody array

The human EGFR phosphorylation antibody array was obtained from RayBiotech (Peachtree Corners, GA, USA) and performed according to manufacturer’s instructions. The array is a dot-blot-based assay specifically designed to detect 17 different phosphorylation sites of the EGFR family, which includes four family members: EGFR, ErbB2, ErbB3, and ErbB4. Briefly, cell lysates obtained from Raji stimulated or not with refp17, EGF, NHL-a101 and NHL-a102 vp17s (0.1 μg/ml) for 5 min were added to antibody array membranes. Then, the membranes were washed and incubated with a cocktail of biotin-conjugated anti-EGFR. After incubation with HRP-streptavidin, the signals were visualized by chemiluminescence. Finally, the density of the immunoreactive dots was acquired by ChemiDoc-it System (Bio-Rad, Hercules, CA, USA) and protein phosphorylation and expression level was analyzed using Gel-Pro Analyzer 6.0 software (Media Cybernetics, Houston, TX, USA).

### Western blot

Raji cells, starved for 24 hours by serum deprivation (RPMI containing 1 mM L-glutamine and 1 mM sodium pyruvate), were stimulated with 100 ng/ml of EGF for 5, 15, 60 min. Then the cells were lysed in 200 μL of 10 mM Hepes (pH 7.9), 10 mM KCl, 1.5 mM MgCl2, 0.5 mM EGTA, 0.5 mM EDTA, and 0.6% Nonidet P-40, containing phosphatase inhibitors (sodium vanadate, PAO, and sodium fluoride) and a mixture of protease inhibitors (Complete Mini Roche, Hoffmann-La Roche, Basel, Switzerland). Equal amounts of total protein were resolved on a 12% SDS-polyacrylamide gel and electroblotted onto a nitrocellulose membrane. The blots were incubated overnight at 4 °C with mAb to pAkt and total Akt (Cell Signaling Technology, Danvers, MA, USA). The antigen-antibody complex was detected by incubation of the membranes for 1 h at room temperature with peroxidase-coupled goat anti-mouse IgG (Thermo Fisher Scientific, Waltham, MA, USA) and revealed using ECL (Enhanced chemiluminescence) System (Santa Cruz Biotechnology, Dallas, TX, USA). Images were acquired by ChemiDoc-it System and protein expression was determined using Gel-Pro Analyzer 6.0 software.

### B-cell colony formation assay

The assay was performed as previously described [14]. Briefly, Raji and Bjab cells were seeded into a 96-well plate at a dilution of 0.5 cells/well. Plates were incubated for 8 and 12 days, respectively, in complete medium in presence or absence of different molecules, shown in Table 1. Then, the culture plates were analyzed for single colony formation. The colony area was measured (15 colonies/condition) by using Leica Qwin image analysis software. The same number of colonies (15 colonies/condition) was aseptically harvested from 96-well plates and stained with propidium iodide (PI) to detect PI-viable cells by flow cytometry. Absolute cell counts were obtained by the counting function of the MACSQuant® Analyzer (Miltenyi Biotec, Bergish Gladbach, Germany).

**Table 1:** Different molecules used in B-cell colony assays.

Reagent	Abbreviation	Final concentration	Role	Supplier
Epidermal growth factor	EGF	100 ng/ml	–	R&D Systems, Minneapolis, MN, USA
Sphingosine-1-phosphate	S1P	100 nM	–	Sigma-Aldrich, St. Louis, MO, USA
Endothelin-1	ET-1	100 nM	–	Sigma-Aldrich, St. Louis, MO, USA
Thrombin	Thr	2 U/ml	–	GE Healthcare, Chicago, IL, USA
AG1478	–	250 nM	EGFR inhibitor	Selleckchem, Houston, TX, USA
AG879	–	2 μM	ErbB2 inhibitor	Selleckchem, Houston, TX, USA
Wortmannin	WT	100 nM	PI3K inhibitor	Enzo, Farmingdale, NY, USA
Akt inhibitor VIII	Akt VIII i	1 μM	Akt inhibitor VIII	Sigma-Aldrich, St. Louis, MO, USA
PD98059	–	10 μM	Mitogen-activated protein kinase (MEK)/ERK1/2 inhibitor	Merck Life Science, Milano, Italy
YM-254890	–	100 nM	Gα_q_ inhibitor	Adipogen Life Sciences, San Diego, CA, USA
Pertussis toxin	PTX	10 ng/ml	Gi inhibitor	Tocris Bioscience, Bristol, UK
Batimastat	–	20 nM	Metalloprotease (MMP) inhibitor	Tocris Bioscience, Bristol, UK
Ilomastat	–	1 μM	Metalloprotease (MMP) inhibitor	Tocris Bioscience, Bristol, UK
W146	–	100 nM	Selective antagonist of S1PR1	Sigma-Aldrich, St. Louis, MO, USA
JTE-013	–	10 nM	Selective antagonist of S1PR2	Sigma-Aldrich, St. Louis, MO, USA
TY-52156	–	100 nM	Selective antagonist of S1PR3	Sigma-Aldrich, St. Louis, MO, USA
CYM-50358	–	1 nM	Selective antagonist of S1PR4	Sigma-Aldrich, St. Louis, MO, USA
BQ123	–	0.65 μg/ml	Endothelin A receptor antagonist	Sigma-Aldrich, St. Louis, MO, USA
BQ788	–	0.65 μg/ml	Endothelin B receptor antagonist	Sigma-Aldrich, St. Louis, MO, USA
SCH79797	–	10 nM	Selective antagonist of PAR-1	Tocris Bioscience, Bristol, UK
tcy-NH2	–	10 μM	Selective antagonist of PAR-4	Tocris Bioscience, Bristol, UK
Monoclonal antibody to PAR1 (ATAP2, sc-13503)	mAb to PAR1	1 mg/ml	–	Santa Cruz Biotechnology, Dallas, TX, USA

### Production of recombinant GST-linked PAR-1 in a bacterial expression system

The expression and production of PAR1 has been performed as previously described for G protein-coupled receptors (GPCR) [19]. The coding sequence of PAR-1 (accession number: P25116) has been synthetized by Integrated DNA Technologies (IDT; Coralville, Iowa, USA) and cloned into the BamHI and XhoI sites of the prokaryotic expression vector pGEX-4T-1 (GE Healthcare). The glutathione S-transferase (GST) fusion PAR-1 was expressed in BL21 (DE3). Then, bacteria were resuspended in PBS containing 10 mM EDTA, 0.1% (v/v) Triton X-100, 100 μg/ml lysozyme, protease inhibitor cocktail and subjected to 4 freeze/thaw cycles. MgCl_2_ (20 mM) and Triton X-100 (2% v/v) were added and bacterial lysate was centrifuged at 16,000 × *g* for 15 min to separate soluble protein from inclusion bodies. PAR-1 receptor was purified from supernatant using glutathione Sepharose 4B beads and then analyzed for purity by SDS-PAGE and western blot using the anti-PAR-1 mAb (ATAP2).

### Surface plasmon resonance (SPR) binding assay

The interaction of refp17, NHL-a101, NHL-a102, F1 or F4 peptide with PAR1 was carried out as previously described [8, 19]. Briefly, SPR measurements were performed on a BIAcore X100 instrument (GE Healthcare). Anti-GST antibody was immobilized on a CM5 sensor chip (GE Healthcare) using standard amine-coupling chemistry. Then, recombinant human PAR1 with a C-terminal GST tag (GST-PAR1; 10 μg/mL) in running buffer containing 50 mM Hepes (pH 7.0), 0.01% CHS (cholesteryl hemisuccinate Tris salt), 0.1% CHAPS {3-[(3-cholamidopropyl) dimethylammonio]-1-propanesulfonate} and 0.33 mM DOPC:DOPS [synthetic phospholipid blend (dioleoyl) (7:3 wt/wt); Avanti polar lipids] was injected over the anti-GST surface at a flow rate of 5 μL/min, allowing the capture of about 854 Resonance Unit. In the same experimental conditions, the recombinant GST was captured and used as negative control for blank subtraction. The immobilization of the receptor to the sensor chip surface was confirmed by the injection of an anti-PAR1 mAb (ATAP2) in running buffer at a flow rate of 30 μL/min. NHL-a101, NHL-a102 and F1 peptide were injected over PAR1 surface at different concentrations (from 125 to 1000 nM) as described for anti-PAR1 antibody. Kinetic parameters were obtained from sensorgram overlays by using the non-linear fitting (single site model) software package BIAevaluation 3.2. Only sensorgrams with fitting *chi square* values close to 10 were used [20].

### Co-immunoprecipitation assay

Raji cells (1.5 × 10^6^) were incubated with 1.5 μg of NHL-a102 for 15 min at room temperature. After incubation, chemical cross-linker BS3 was added and incubated for 2 h. At the end of incubation, cells were washed with PBS to remove unbound viral protein and solubilized in 200 μl of lysis buffer containing a phosphatase inhibitor cocktail (10 min at 4 °C). Then, the lysates were centrifuged at 10,000 × *g* at 4 °C for 10 min. The supernatants were subjected to a preclearing step with 20 μl protein G Plus-Agarose suspension (Thermo Fisher Scientific) and incubated for 30 min at 4 °C. After brief centrifugation, recovered supernatants were incubated overnight at 4 °C with 10 μg of anti-p17 mAb MBS-34 [21] on a rocker. The next day, 20 μl of protein G Plus-Agarose was added to the samples and incubated for 1 h at 4 °C. The beads were collected by centrifugation, washed with PBS, resuspended in sample buffer (30 μl) and analyzed on SDS-PAGE gel (10%). The immunocomplexes were transferred onto an Immobilon PVDF membrane (Millipore, Bedford, MA) and incubated overnight at 4 °C with an anti-PAR1 mAb [ATAP2 1:500 in TTBS (Tris-HCl 10 mM pH 7.5, NaCl 150 mM, 0.1% Tween 20)]. After incubation at 4 °C for 1 h with peroxidase-coupled goat anti-mouse IgG (diluted 1:5000 in TTBS), antigen-antibody complexes were detected and revealed using ECL System (Santa Cruz Biotechnology). Images were acquired by ChemiDoc-it System.

### Statistical analysis

For each set of experiments the sample size was chosen to ensure adequate power to detect variations. Data obtained from multiple independent experiments are expressed as the means ± the standard deviations (SD). When appropriate data were tested for normality and variance similarity. The data were analyzed for statistical significance using one-way ANOVA. Bonferroni’s post-test was used to compare data. Differences were considered significant at *P* < 0.05. Statistical tests were performed using Prism 8 software (GraphPad, San Diego, CA, USA).

## Results

### NHL-a101 and NHL-a102 vp17s modulate similar signaling pathways promoting cell cycle progression through EGFR

The gain of B cell clonogenic function showed by vp17s may arise from their interaction with an alternate receptor(s) [12]. Activation of different intracellular components of signal transduction is receptor-specific and driven by phosphorylation of critical proteins, which control signaling pathways and cell responses [22]. Therefore, to investigate the possible involvement of an alternate p17 receptor in B-cell clonogenicity triggered by NHL-a101 and NHL-a102, we evaluated the intracellular signaling phosphorylation network in B cells by employing a high-throughput antibody array technology. Raji lymphoma cells were stimulated or not with refp17, NHL-a101, and NHL-a102 (100 ng/ml) and the lysates were processed and analyzed by Kinex™ KAM-850 Antibody Microarray from Kinexus Bioinformatics Corporation, which recognizes different epitopes on 466 proteins. To rule out false positive signals, relevant phosphorylations and up- or down-modulated proteins were confirmed by a multi-immunoblot analysis performed by Kinexus Bioinformatics Corporation. Table 2 shows the proteins confirmed by immunoblot and signals relative to NHL-a101 and NHL-a102 stimulation, normalized to refp17. Literature data mining and Gene Ontology biological process database highlighted that most of these proteins, observed in the immunoblot, as RAC1, ABL1, p53, CDK1, NPM, Rb, PTP-1B, and STAT1, are involved in cell cycle regulation and cancer progression.

**Table 2:** Effect of NHL-a101 and NHL-a102 on expression and phosphorylation levels of intracellular signaling proteins.

Protein short name	Uniprot	Protein name	Phosphorylation site	NHL-a101 vs p17 (%)	NHL-a102 vs p17 (%)
ABL1	P00519	Tyrosine-protein kinase ABL1	Y393	+44%	+123%
CDK1	P06493	Cyclin-dependent kinase 1	T161	+60%	+35%
CDK2	P24941	Cyclin-dependent kinase 2	–	+2%	+54%
CDK4	P11802	Cyclin-dependent kinase 4	–	+27%	−62%
CDK6	Q00534	Cyclin-dependent kinase 6	–	+52%	+10%
CHEK1	O14757	Serine/threonine-protein kinase Chk1	S280	+18%	−38%
CHEK2	O96017	Serine/threonine-protein kinase Chk2	–	−20%	−2%
EGFR	P00533	Epidermal growth factor receptor	–	−5%	−54%
EGFR	P00533	Epidermal growth factor receptor	T693	−68%	−94%
EGFR	P00533	Epidermal growth factor receptor	Y998	+12%	−86%
eIF-2A	P19525	Interferon-induced, double-stranded RNA-activated protein kinase	T446	−69%	−117%
hMSH2	P43246	DNA mismatch repair protein Msh2	–	+42%	+216%
JAK1	P23458	Tyrosine-protein kinase JAK1	Y1034	+22%	−208%
MAPK 8	P45983	Mitogen-activated protein kinase 8	–	−11%	+31%
NPM	P06748	Nucleophosmin	S4	+97%	+22%
p53	P04637	Cellular tumor antigen p53	–	−11%	+0%
p53	P04637	Cellular tumor antigen p53	S33	−6%	−127%
PTP-1B	P18031	Tyrosine-protein phosphatase non-receptor type 1	–	+176%	+1095%
RAC1	P63000	Ras-related C3 botulinum toxin substrate 1	–	+11%	+30%
RAF1	P23458	Tyrosine-protein kinase JAK1	S259	−6%	−346%
Rb	P06400	Retinoblastoma-associated protein	S780	−25%	+111%
Rb	P06400	Retinoblastoma-associated protein	S795	+55%	+111%
STAT1	P42224	Signal transducer and activator of transcription 1-alpha/beta	S272	+27%	+17%

The protein expression and phosphorylation level were analyzed from Kinexus Bioinformatic Corporation by densitometric analysis of multi-immunoblotting. Blot quantification data of vp17s were compared to refp17 treated samples for the interpretation of the results. The numerical values refer to vp17/refp17 ratio and are expressed as percentage.

**Fig. 1:**
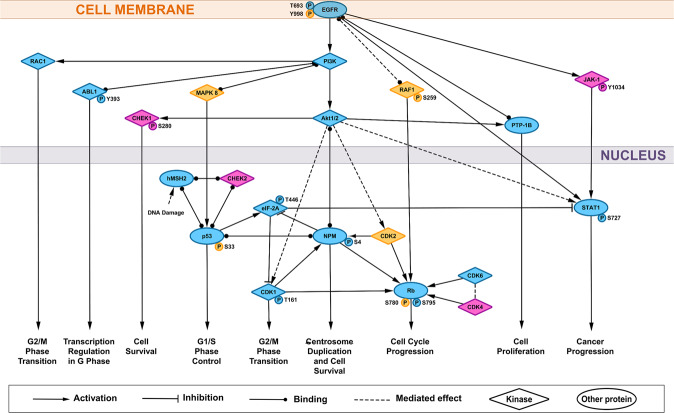
Representation of the signaling pathways involved in B-cell clonogenicity induced by clonogenic vp17s. Stimulation of B cells with NHL-a101 and NHL-a102 vp17s induces the activation of several signaling molecules involved in promoting cell survival, cell proliferation, cell cycle progression, G1/S and G2/M phase transition. STRING database and literature data mining were used to identify known and experimentally verified interactions. The several kinases involved in the pathway are represented by diamonds, the other proteins by ellipses. The proteins represented in cyan are the ones activated by both NHL-a101 and NHL-a102. The molecules activated by NHL-a101 are in magenta, the ones stimulated by NHL-a102 in yellow. PI3K: phosphatidylinositol-3-kinase; Akt: protein kinase B; CDK1: cyclin-dependent kinase 1; ABL1: tyrosine-protein kinase ABL1; CDK2: cyclin-dependent kinase 2; CDK4: cyclin-dependent kinase 4; CDK6: cyclin-dependent kinase 6; CHEK1: serine/threonine-protein kinase Chk1; CHEK2: serine/threonine-protein kinase Chk2; EGFR: epidermal growth factor receptor; eIF-2A: interferon-induced, double-stranded RNA-activated protein kinase; hMSH2: DNA mismatch repair protein Msh2; JAK-1: tyrosine-protein kinase JAK1; MAPK 8: mitogen-activated protein kinase 8; NPM: nucleophosmin; p53: cellular tumor antigen p53; PTP-1B: tyrosine-protein phosphatase non-receptor type 1; RAC1: ras-related C3 botulinum toxin substrate 1; RAF1: tyrosine-protein kinase JAK1; Rb: Retinoblastoma-associated protein; STAT1: signal transducer and activator of transcription 1-alpha/beta.

The STRING database is a protein–protein interaction meta-database, which combines multiple databases to provide protein–protein interactions, based on experimental data, literature mining, curated databases and other sources [18]. Our set of proteins was used as input for STRING to identify critical protein–protein interactions and design the signaling pathways activated by vp17s. The pertinence of the STRING molecular interactions was then verified with literature data mining on multiple databases (PubMed, PhosphoSite, Uniprot, and Genecards). This bioinformatic analysis highlighted EGFR as the receptor triggered by both vp17s together with the activation of the PI3K/Akt signaling pathway, consistently with previous data [12, 23] (Fig. 1). The comparison between the signaling proteins triggered by the two vp17s allowed to also identify the contribution of other proliferation-regulating molecules specific for each variant: CHEK1, CHEK2, JAK1, and CDK4, for NHL-a101; MAPK8, RAF1, and CDK2 for NHL-a102.

Overall, these results suggest an involvement of the EGFR signaling in mediating vp17s clonogenicity through the modulation of several molecules involved in cancer progression.

### Clonogenic vp17s induce EGFR activation

**Fig. 2:**
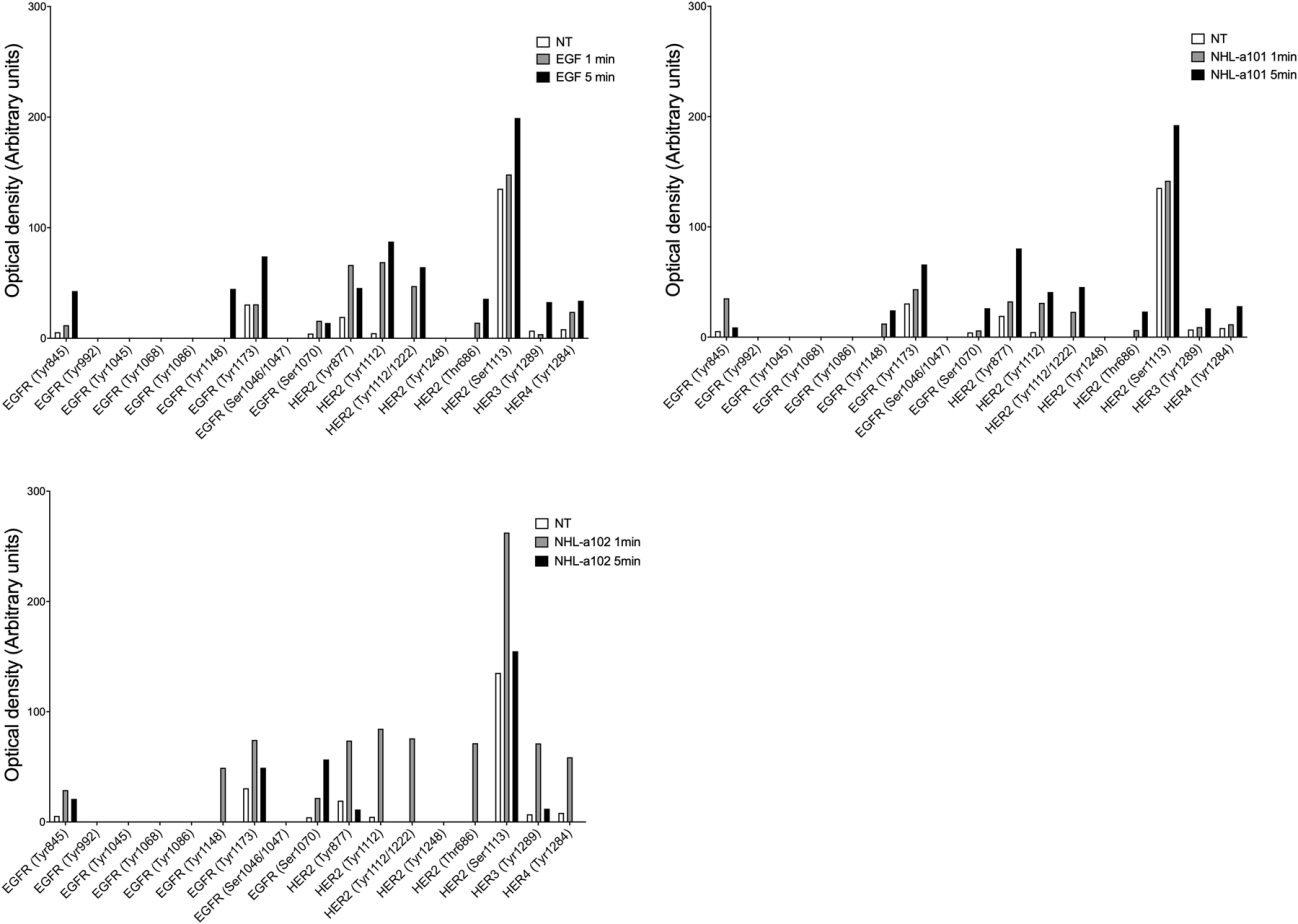
EGFR activation by vp17s in Raji B cells. Cellular extracts of Raji B cells treated for 5 min at 37 °C with or without EGF, NHL-a101 and NHL-a102 were evaluated for phosphorylation of EGFR family members by a human phosphorylation array. The intensities of the phospho-protein signals were quantified by densitometric analysis and normalized to either positive controls or not treated cells as suggested by the manufacturer’s instructions. Values reported for protein phosphorylation levels are representative of one representative experiment of 3 with similar results. NT, not treated.

To verify vp17-triggered EGR activation, we performed experiments using the RayBio human EGFR phosphorylation antibody array, which simultaneously detects 17 different specific phosphorylation sites on EGFR family members. Raji cells, which basally express EGFR [24], were stimulated or not for 1 and 5 min with NHL-a101 and NHL-a102 (100 ng/ml) and then lysed to obtain cellular extracts. Cells stimulated with EGF (100 ng/ml) were used as positive control. As shown in Fig. 2, Raji stimulated with EGF or the two vp17s, as compared to untreated cells, showed an increase in EGFR phosphorylation levels at Tyr845, Tyr1148, Tyr1173, Ser1070 residues, and in ErbB2 ones at Tyr877, Tyr1112, Tyr1112/1222, Thr686, and Ser1113. The two vp17s showed a different kinetics of receptor phosphorylation. While the phosphorylation kinetic triggered by NHL-a101 at 1 and 5 min appeared superimposable to that promoted by EGF with a peak at 5 min, NHL-a102 triggered a peak of receptor phosphorylation at 1 min only. These findings confirm that both vp17s are able to activate EGFR and suggested the possibility that these variants could trigger not only the formation of EGFR homodimers, but also heterodimers with the ErbB2 member of the EGFR family [25].

### B-cell clonogenic activity of vp17s is mediated by EGFR and ErbB2

**Fig. 3:**
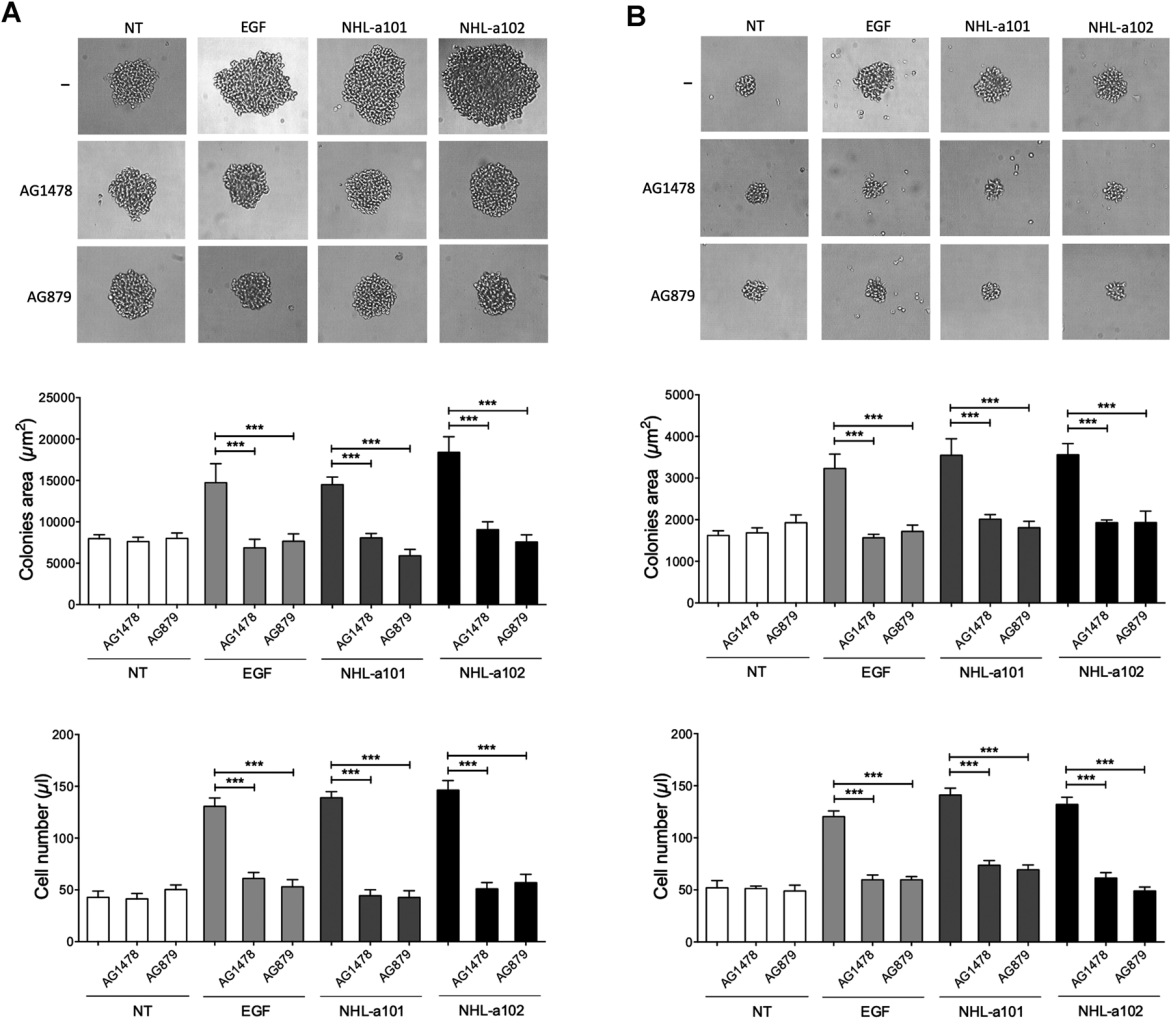
Effect of EGFR and ErbB2 inhibitors on clonogenic activity of vp17s in B-cells. **A**, **B** Raji (**A**) and Bjab (**B**) cells were cultured for 8 days and 12 days, respectively, in the presence or absence of EGF (100 ng/ml) or NHL-a101 or NHL-a102 (10 ng/ml) and EGFR inhibitor AG1478 (250 nM) or ErbB2 inhibitor AG879 (2 μM). Bright-field images represent the characteristic morphology of 2D colonies of Raji and Bjab (upper panels), one colony for each condition is shown (original magnification, ×40). The colony area was measured (15 colonies/condition) by using Leica Qwin image analysis software (central panel). The same number of colonies (15 colonies/condition) was aseptically harvested from 96-well plates and stained with propidium iodide (PI) to detect PI-viable cells by flow cytometry (lower panel). Absolute cell counts were obtained by the counting function of the MACSQuant® Analyzer. Bars represent the means ± SD of three independent experiments. The statistical significance between control and treated cultures was calculated using one-way ANOVA and the Bonferroni’s post-test was used to compare data. NT, not treated. ***P < 0.001.

Phosphoproteomic analysis and phosphorylation antibody array on cell lysates, obtained from B-cells stimulated with NHL-a101 and NHL-a102, showed EGFR and ErbB2 phosphorylation (Table 2 and Fig. 2). To assess whether the clonogenic activity of vp17s is mediated by EGFR and ErbB2, we utilized a single cell cloning assay [14]. This method was performed by seeding a single B-cell in each well of a 96-well plate in the presence or absence of EGF (100 ng/ml) or NHL-a101 or NHL-a102 (10 ng/ml) and the selective EGFR inhibitor AG1478 (250 nM) or the ErbB2 inhibitor AG879 (2 μM). At day 8 of culture, Raji cells formed a visible single colony in >60% of seeded wells attesting for active cell proliferation (Fig. 3A, upper panel). As expected, NHL-a101 and NHL-a102, as well as EGF, promoted the formation of colonies with a significantly larger size than those cultured in medium alone. The presence of both AG1478 and AG879 selective inhibitors completely blocked the clonogenic activity of EGF and vp17s (Fig. 3A, central panel, left). A cell suspension was then obtained by pooling equal number of colonies per each experimental condition and the absolute number of cells was evaluated by propidium iodide staining and flow cytometry. As shown in Fig. 3A (lower panel, left), the vp17s significantly increased B-cell proliferation, as compared to untreated cells, an effect that was completely inhibited by AG1478 and AG879 inhibitors.

Raji is an Epstein-Barr virus (EBV)-infected human lymphoma B-cell line. Therefore, in order to verify the involvement of EGF and ErbB2 receptors in vp17s clonogenic activity independently of EBV protein expression, we also tested the inhibition of vp17-triggered cell proliferation by AG1478 and AG879 in the EBV-negative BJAB human lymphoma B-cell line. As for Raji, NHL-a101 and NHL-a102 significantly increased cell proliferation as compared with untreated control cultures, whereas the presence of both AG1478 and AG879 significantly reduced their clonogenic activity (Fig. 3B). Overall, these results confirm the involvement of EGFR and ErbB2 in vp17s-induced B-cell growth, irrespective of the presence of EBV.

### EGFR-activated PI3K/Akt signaling pathway promotes vp17-triggered B-cell clonogenicity

**Fig. 4:**
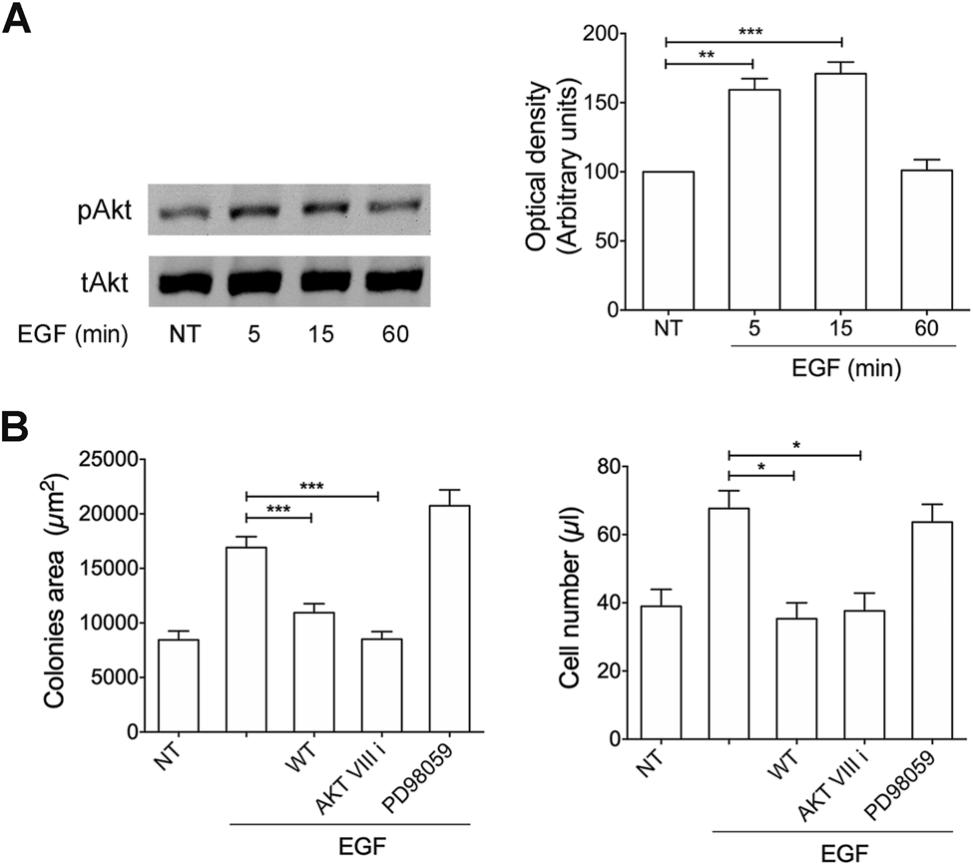
EGFR triggering is linked to PI3K/Akt signaling pathway activation. **A** Raji cells were stimulated with 100 ng/ml of EGF at 37 °C for the indicated times. Not treated cells (NT) were used as control (lane 1). Western blot analysis of Raji lysates shows that EGF ligand activates Akt, as shown by the respective phosphorylation state, verified by densitometric analysis and plotting of the phospho-Akt/total Akt (pAkt/tAkt). In the left panel blots from one representative experiment of 3 with similar results are shown. In the right panel, values reported for Akt phosphorylation are the mean ± SD of three independent experiments. Statistical analysis was performed by one-way ANOVA and the Bonferroni’s post-test was used to compare data. **B** Raji cells were cultured for 8 days in the presence or absence of EGF (100 ng/ml) and PI3K inhibitor WT (100 nM) or Akt inhibitor VIII (1 μM) or MEK/ERK1/2 inhibitor PD98059 (10 μM) The colony area was measured (15 colonies/condition) by using Leica Qwin image analysis software (left panel). The same number of colonies (15 colonies/condition) was aseptically harvested from 96-well plates and stained with propidium iodide (PI) to detect PI-viable cells by flow cytometry. Absolute cell counts were obtained by the counting function of the MACSQuant® Analyzer (right panel). Bars represent the means ± SD of three independent experiments. The statistical significance between control and treated cultures was calculated using one-way ANOVA and the Bonferroni’s post-test was used to compare data. *P < 0.05; **P < 0.01; ***P < 0.001.

To confirm that clonogenicity is related to activation of the PI3K/Akt pathway mediated by EGFR, we first explored the capability of EGF to modulate Akt phosphorylation. Raji cells stimulated for 5, 15 and 60 min with EGF (100 ng/ml) showed a significant increase in the levels of Akt phosphorylation, compared to untreated cells (Fig. 4A). We also performed single cell cloning assays in the presence or absence of EGF (100 ng/ml) and wortmannin (WT, 100 nM) or Akt inhibitor VIII (1 μM), which inhibit PI3K and Akt, respectively. The B-cell growth-promoting activity of EGF was significantly inhibited by both inhibitors (Fig. 4B, left panel). Moreover, the cell suspension obtained by pooling equal number of colonies per each experimental condition and the evaluation of absolute number of cells confirmed the inhibition of vp17s-induced proliferation by WT and Akt inhibitor VIII (Fig. 4C, right panel). Notably, the activity of EGF on B cell proliferation was not modulated by an inhibitor of MEK/ERK1/2 (PD98059, 10 μM), used as negative control, confirming the specificity of our findings. These data confirm that EGFR-induced B cell clonogenicity is linked to activation of the PI3K/Akt signaling pathway.

### B-cell clonogenic activity of vp17s occurs through the G protein-coupled receptors-mediated EGFR transactivation

**Fig. 5:**
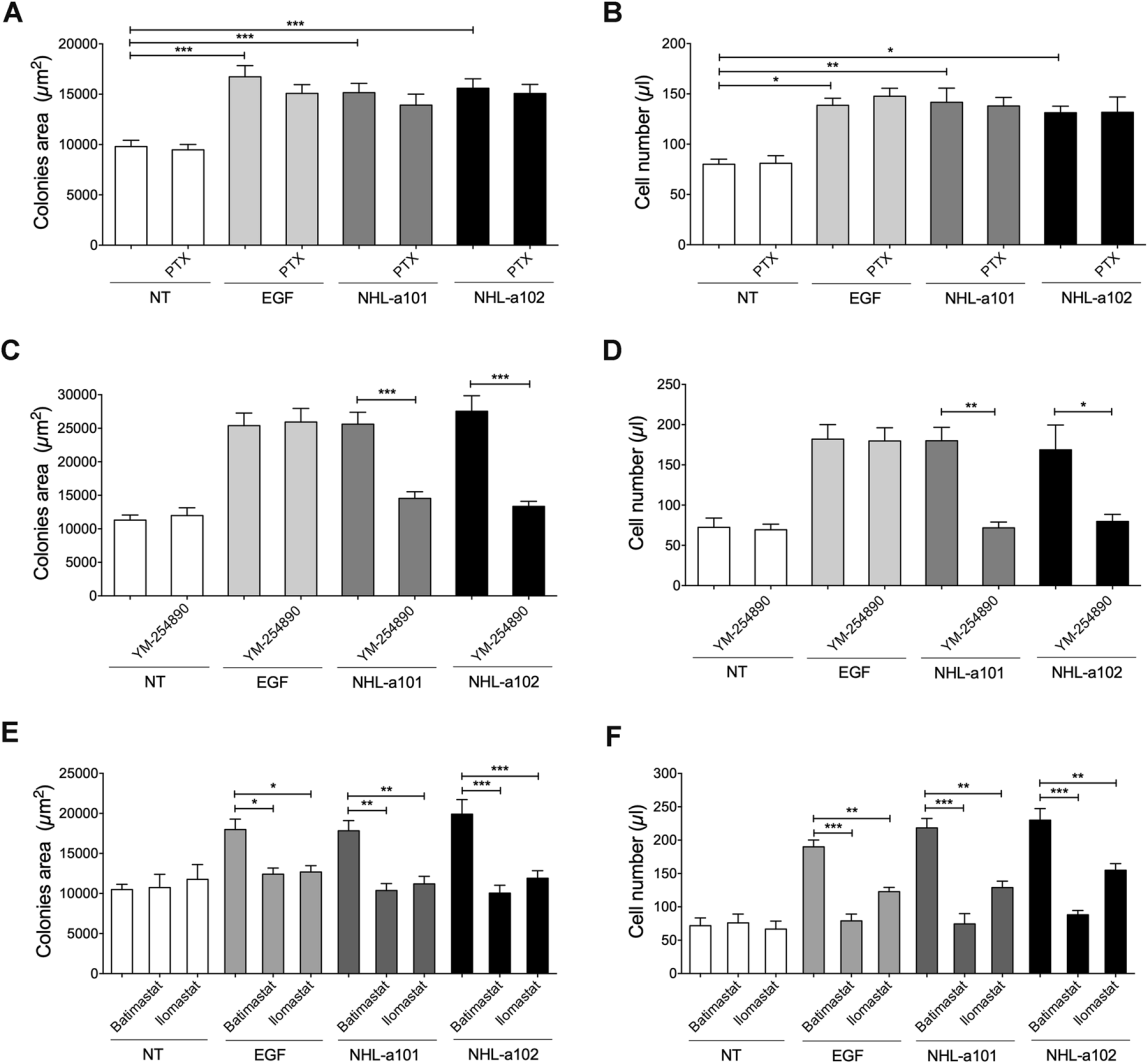
Effect of Gi, Gq and MMPs inhibitors on vp17s-induced B-cell clonogenic activity. **A**, **C**, **E** Raji cells were cultured for 8 days in the presence or absence of EGF (100 ng/ml) or NHL-a101 or NHL-a102 (10 ng/ml) and pertussis toxin (PTX; 10 ng/ml) (**A**) or YM-254890 (100 nM) (**C**) or Batimastat and Ilomastat (**E**). The colony area of Raji was measured (15 colonies/condition) by using Leica Qwin image analysis software. **B**, **D**, **F** The same number of colonies (15 colonies/condition) was aseptically harvested from 96-well plates and stained with propidium iodide (PI) to detect PI-viable cells by flow cytometry. Absolute cell counts were obtained by the counting function of the MACSQuant® Analyzer. Bars represent the means ± SD of three independent experiments. The statistical significance between control and treated cultures was calculated using one-way ANOVA and the Bonferroni’s post-test was used to compare data. NT , not treated. *P < 0.05; **P < 0.01; ***P < 0.001.

We previously demonstrated that the different p17 activities are promoted by interactions of the protein with G-coupled receptors. Transactivation of EGFR occurs by different GPCR through different mechanisms [26]. In particular, GPCRs ligands may exert their effects on EGFR by activating the heterotrimeric G proteins, particularly Gi and Gq, which are mainly involved in regulation of cell proliferation. To assess the involvement of these G proteins in GPCR-induced EGFR transactivation in vp17s clonogenicity, we performed single cell cloning assays with Raji cells in presence of NHL-a101 or NHL-a102 (10 ng/ml) and pertussis toxin (PTX; 10 ng/ml) or YM-254890 (100 nM), which block selectively Gi- and Gq-mediated signaling, respectively. The vp17s-triggered proliferation of Raji cells was completely insensitive to PTX treatment (Fig. 5A), whereas the Gq inhibitor YM-254890 significantly blocked the clonogenic activity of both vp17s (Fig. 5B). The evaluation of absolute number of cells forming the colonies confirmed the proliferation inhibition by YM-254890, but not by PTX (Figs. 5D and C, respectively).

These results highlight the involvement of EGFR transactivation from a GPCR via Gq protein activation in vp17s-induced B-cell clonogenicity.

### The B-cell clonogenicity induced by vp17s is sustained by a membrane-bound matrix metalloproteases (MMPs)-dependent loop

EGFR transactivation by GPCRs was shown to be MMP-dependent [27]. Indeed, stimulation of GPCRs leads to MMP activation, resulting in the shedding of biologically active soluble ligands, which in turn activate EGFR and its downstream signaling cascades [28]. In some cases, MMP activation has been also shown to occur downstream EGFR transactivation through modulation of gene expression [29].

The involvement of MMPs in GPCR-mediated EGFR transactivation by vp17s was investigated by performing single cell cloning assays with Raji cells in presence of NHL-a101 or NHL-a102 (10 ng/ml) and two different broad-spectrum metalloprotease inhibitors, Batimastat (20 nM) or Ilomastat (1 μM). Both MMP inhibitors had a noticeable inhibitory effect on the clonogenic activity of vp17s (Fig. 5E). On the other hand, selective inhibition of MMPs by both inhibitors significantly diminished also EGF-induced clonogenicity, thus suggesting a positive feedback exerted by EGF-induced MMPs expression. Propidium iodide staining and flow cytometry confirmed the inhibition of EGF-induced cell proliferation by Batimastat and Ilomastat (Fig. 5F). These results are consistent with the notion that MMP-dependent ligand-mediated EGFR signaling plays an important role in regulation of cell proliferation in a variety of cancer types [30] and suggest that vp17s-induced EGFR transactivation promotes downstream signaling pathways, which drive its progressive induction through an autocrine feedback mechanism.

**Fig. 6:**
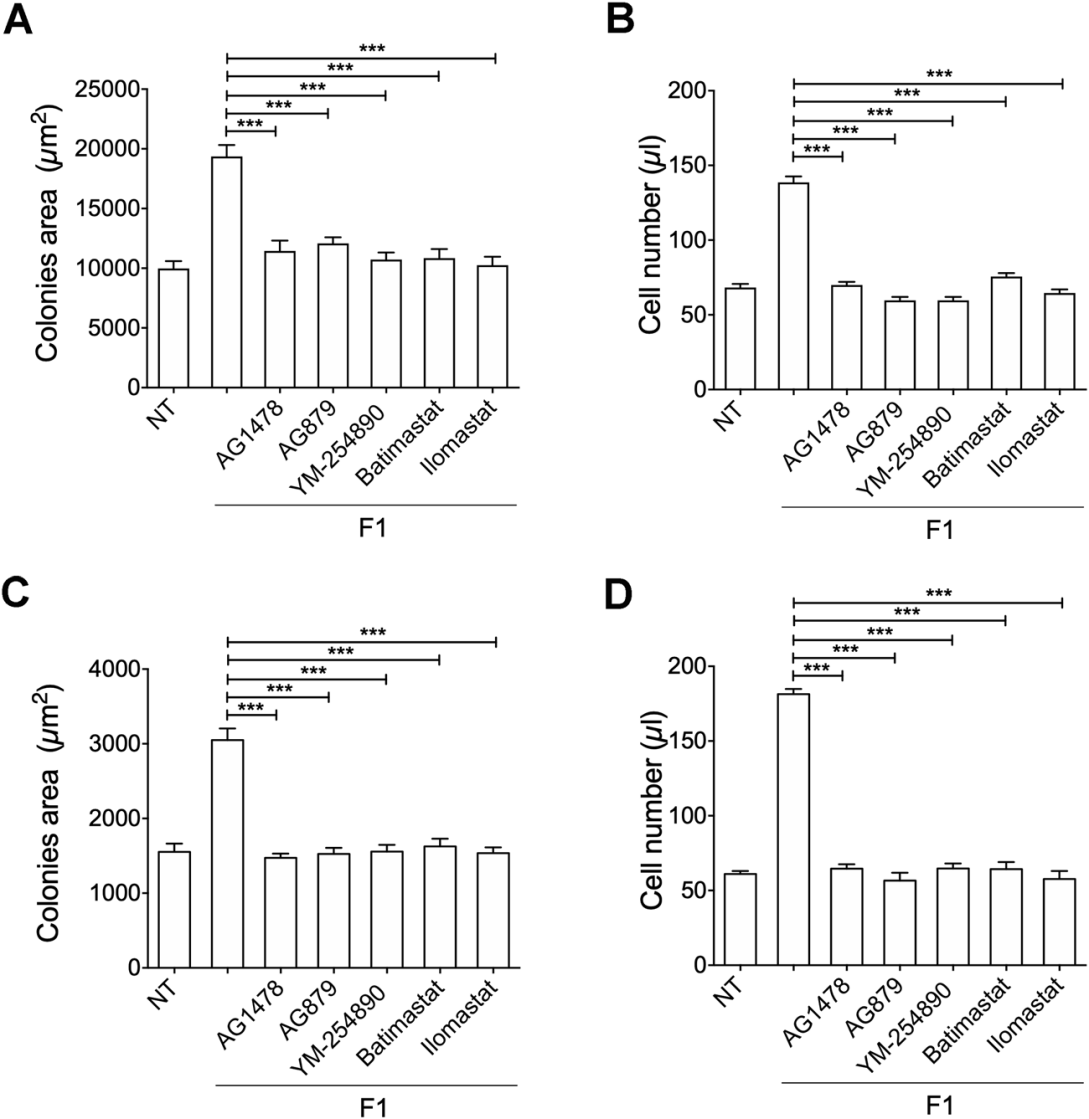
Effect of EGFR, ErbB2, Gq, and MMPs inhibitors on F1-induced B-cell clonogenicity. **A**, **C** Raji (**A**) and Bjab (**C**) cells form colonies when sorted into 96-well plates as single cell. Plates were cultured for 8 and 12 days, respectively, in the presence or absence of F1 peptide (10 ng/ml) and EGFR inhibitor AG1478 (250 nM) or ErbB2 inhibitor AG879 (2 μM) or YM-254890 (100 nM), which blocks Gq-mediated signaling. The colony area of Raji (**A**) and Bjab (**C**) was measured (15 colonies/condition) by using Leica Qwin image analysis software (left panel). **B**, **D** The same number of colonies (15 colonies/condition) was aseptically harvested from 96-well plates and stained with propidium iodide (PI) to detect PI-viable cells by flow cytometry. Absolute cell counts were obtained by the counting function of the MACSQuant® Analyzer. Bars represent the means ± SD of three independent experiments. The statistical significance between control and treated cultures was calculated using one-way ANOVA and the Bonferroni’s post-test was used to compare data. NT, not treated. ***P < 0.001.

Functional mapping of refp17 domains contributed to identify the N-terminal sequence (peptide F1: GARASVLSGGELDRWEKIRL), spanning from aa 2 to 21, as the p17 functional epitope responsible for PI3K/Akt activation and B-cell clonogenicity [14]. To assess whether the F1 peptide acts as the whole NHL-a101 and NHL-a102 clonogenic proteins through EGFR transactivation via Gq protein and MMP activity, we performed single cell cloning assays with Raji and BJAB cells in presence or absence of F1 peptide (10 ng/ml) and EGFR inhibitor AG1478 (250 nM), ErbB2 inhibitor AG879 (2 μM), Gq inhibitor YM-254890 (100 nM), MMP inhibitors, Batimastat (20 nM) or Ilomastat (1 μM). As shown in Fig. 6A and C, the presence of AG1478, AG879, YM-254890, Batimastat and Ilomastat significantly blocked the clonogenic activity of F1 peptide in Raji and BJAB cells, respectively, as well as F1-promoted Raji and BJAB cell proliferation (Figs. 6B and D, respectively). These data confirm the capability of F1 peptide alone to exploit the same molecular interactions and receptor engagement of the clonogenic vp17s to induce B cell clonogenicity.

### B-cell clonogenicity induced by vp17s is triggered by protease-activated receptors (PARs)

**Fig. 7:**
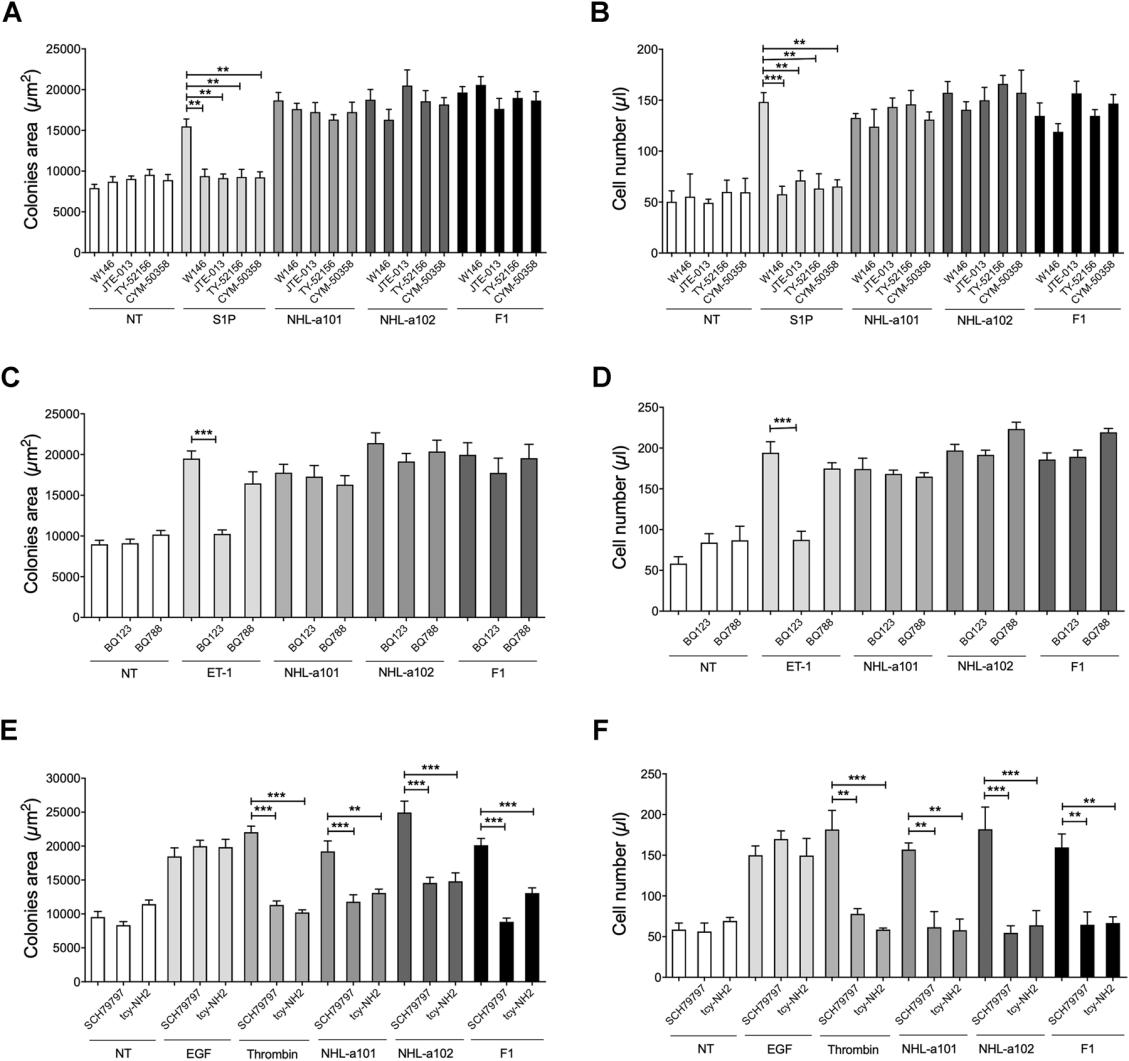
Effect of specific inhibitors for S1PRs, ETRs, and PARs on vp17s-induced B-cell clonogenicity. **A**, **C**, **E** Raji cells were cultured for 8 days in the presence or absence of NHL-a101 or NHL-a102 (10 ng/ml) or **A** agonist S1P (100 nM) and selective antagonists W146 (100 nM), JTE-013 (10 nM), TY-52156 (100 nM), CYM-50358 (1 nM) for S1PR1, S1PR2, S1PR3, S1PR4, respectively, or **C** agonist ET-1 (100 nM) and ETA and ETB receptor antagonist BQ123 or BQ788 (0.65 μg/ml), respectively, or **E** EGF (used as negative control), thrombin (2 U/ml) and selective antagonist SCH79797 (10 nM) for PAR1 and tcy-NH2 (10 μM) for PAR4. The colony area of Raji was measured (15 colonies/condition) by using Leica Qwin image analysis software. **B**, **D**, **F** The same number of colonies (15 colonies/condition) was aseptically harvested from 96-well plates and stained with Propidium Iodide (PI) to detect PI-viable cells by flow cytometry. Absolute cell counts were obtained by the counting function of the MACSQuant® Analyzer. Bars represent the means ± SD of three independent experiments. The statistical significance between control and treated cultures was calculated using one-way ANOVA and the Bonferroni’s post-test was used to compare data. NT, not treated. **P < 0.01; ***P < 0.001.

EGFR transactivation has been particularly well studied for GPCR agonists, such as sphingosine-1-phosphate (S1P), endothelin-1 (ET-1) and thrombin, which induce cleavage of EGF-like precursors, leading to phosphorylation of EGFRs [31]. Single cell cloning assay was used to assess whether the vp17 clonogenic activity is triggered by sphinghosine-1-phosphate receptors (S1PRs), endothelin receptors (ETRs) or protease-activated receptors (PARs), in particular PAR1 and PAR4, which are known to affect tumorigenesis [32]. The assay was performed in the presence or absence of the S1P agonist (100 nM) and its selective antagonists W146 (100 nM), JTE-013 (10 nM), TY-52156 (100 nM), CYM-50358 (1 nM) for S1PR1, S1PR2, S1PR3, S1PR4, respectively (Fig. 7A and B), of ET-1 (100 nM) and its ETA and ETB receptor antagonist BQ123 or BQ788 (0.65 μg/ml), respectively (Fig. 7C and D), and of EGF, thrombin (2 U/ml) and its selective antagonist SCH79797 (10 nM) for PAR1 and tcy-NH2 (10 μM) for PAR4 (Figs. 7E and F). Raji cells were stimulated for 8 days with 10 ng/ml of NHL-a101, NHL-a102 or F1 peptide or EGF (100 ng/ml). As expected, in presence of the sole agonist S1P or ET-1 or thrombin, Raji formed visible single colonies with a significantly larger size than untreated cells (NT). As shown in Fig. 7A and C, the presence of selective antagonists for S1PRs or ETRs significantly blocked the clonogenic activity of their respective physiological ligands, but not that induced by vp17s and F1 peptide. Notably, the presence of both PAR selective antagonists SCH79797 and tcy-NH2 significantly and specifically inhibited the clonogenicity induced by the physiological ligand thrombin, vp17s and F1 peptide, but not that induced by EGF, which was used as negative control (Fig. 7E). The evaluation of absolute number of cells forming the colonies per each experimental condition confirmed the inhibition of vp17- and F1-triggered B-cell proliferation by SCH79797 and tcy-NH2 (Fig. 7F), but not by the antagonists for S1PRs or ETRs (Fig. 7B and D, respectively).

Overall, these results suggest that B-cell growth and clonogenicity are triggered by both PAR1 and PAR4, which are known to form heterodimers [33].

### The B-cell clonogenicity of vp17s is mediated specifically by PAR1

**Fig. 8:**
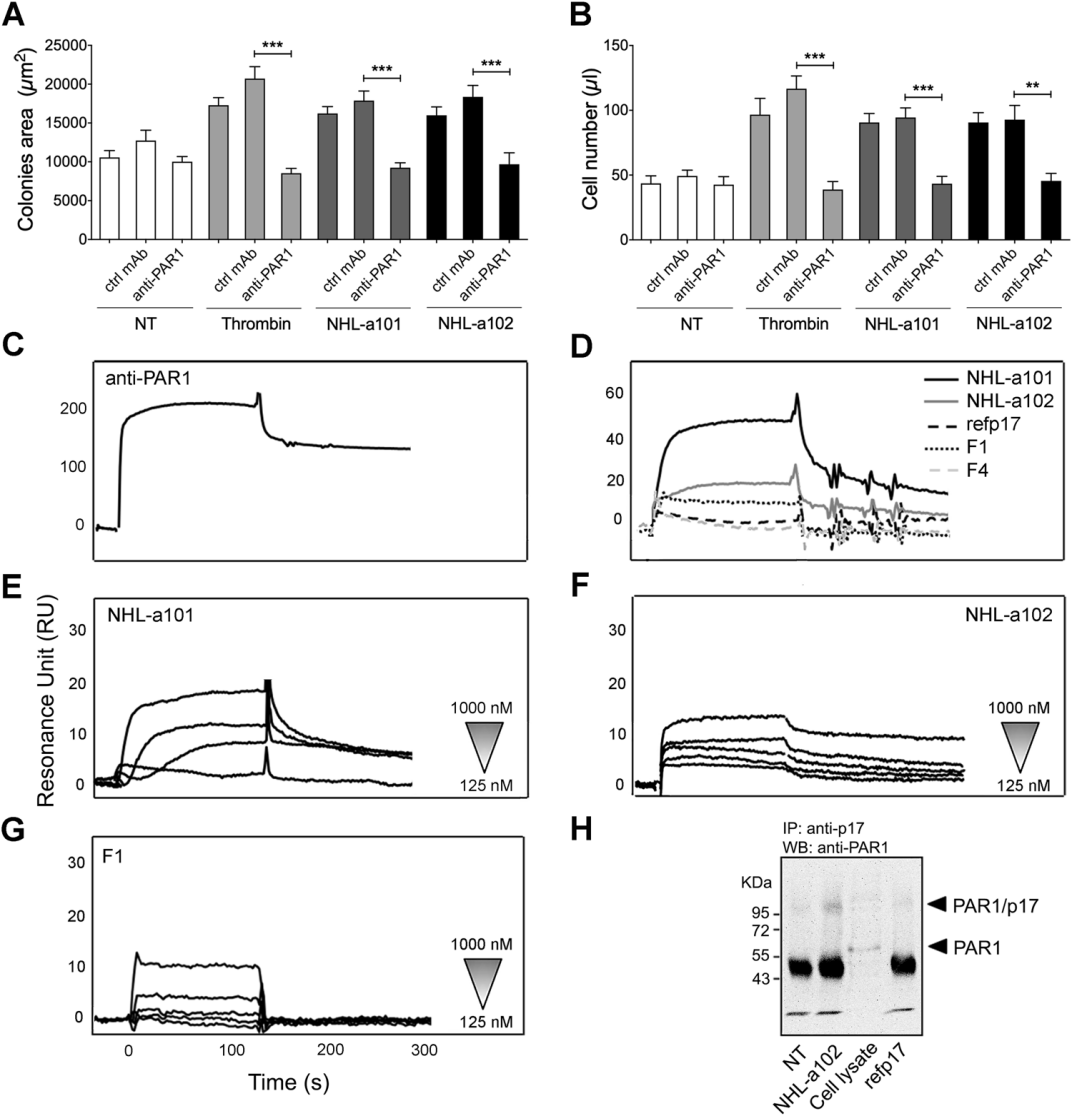
The vp17s-induced B-cell clonogenic activity is mediated by their interaction with PAR1. **A** Raji cells were cultured for 8 days in the presence or absence of NHL-a101 or NHL-a102 (10 ng/ml) or thrombin (2 U/ml) and neutralizing mAb ATAP2 to PAR1 (anti-PAR1) or isotype control mAb (Ctrl mAb; 1 μg/ml). The colony area of Raji was measured (15 colonies/condition) by using Leica Qwin image analysis software. **B** The same number of colonies (15 colonies/condition) was aseptically harvested from 96-well plates and stained with propidium iodide (PI) to detect PI-viable cells by flow cytometry. Absolute cell counts were obtained by the counting function of the MACSQuant® Analyzer. Bars represent the means ± SD of three independent experiments. The statistical significance between control and treated cultures was calculated using one-way ANOVA and the Bonferroni’s post-test was used to compare data. NT, not treated. **P < 0.01; ***P < 0.001. **C**–**G** SPR analysis of the interaction of vp17s and F1 peptide with PAR1. **C** Blank subtracted sensorgram showing the binding of anti-PAR1 mAb (ATAP2). **D** Representative blank subtracted sensorgrams of refp17, NHL-a101, NHL-a102, F1, and F4 binding. **E**, **F**, **G** Overlay of blank subtracted sensorgrams, resulting from the injection of increasing concentration (from 125 to 1000 nM) of NHL-a101 (**E**), NHL-a102 (**F**) and F1 (**G**), used to determinate the kinetic parameter of interaction. RU, resonance unit. **H** Co-immunoprecipitation of p17/PAR1 complexes. Raji cells were incubate or not for 15 min at room temperature with 1.5 μg of vp17 NHL-a102 or refp17. After chemical cross-linking, PAR1 was immunoprecipitated from the lysates with an anti-p17 antibody (MBS-34). The immunoprecipitates were detected by western blotting using a mAb to PAR1 (ATAP2). One representative experiment of three with similar results is shown. NT, not treated.

In order to confirm that vp17-triggered clonogenic activity is mediated by PARs, we performed single cell cloning assays in presence or absence of a neutralizing mAb to PAR1. The assays were performed by stimulating Raji cells for 8 days with NHL-a101 or NHL-a102 (10 ng/ml) or with thrombin (2 U/ml), used as positive control, in the presence or absence of the neutralizing mAb ATAP2 to PAR1 or isotype control mAb (Ctrl mAb; 1 μg/ml). As expected, in the presence of thrombin or vp17s, Raji formed visible single colonies with a significantly larger size than untreated cells (Fig. 8A). The presence of mAb to PAR1 significantly blocks not only the clonogenic activity induced by its respective physiological ligand, but also that triggered by vp17s (Fig. 8A). No inhibition of B-cell clonogenicity induced by thrombin or vp17s was observed in cells treated with control mAb. These data were further confirmed by evaluating the number of cells forming colonies (Fig. 8B).

Surface plasmon resonance (SPR) was then exploited to analyze the capability of vp17s to bind human recombinant PAR1, immobilized on CM5 sensor chip coated with a lipid bilayer through a GST tag, which contributes to the proper tridimensional conformation of the GPCR. The specificity of the surface and the receptor conformation were verified using an anti-PAR1 mAb (ATAP2) (Fig. 8C). Refp17 and F4 peptide (corresponding to aa 47–66 of matrix protein), used as negative controls, did not bind to PAR1, whereas NHL-a101, NHL-a102, and the F1 peptide were able to bind the chip-immobilized receptor (Fig. 8D). In order to determine the kinetic parameters of interactions, the vp17s and F1 peptide were injected at different concentrations (from 125 to 1000 nM) on surface-immobilized PAR1. Either vp17s or F1 peptide bound to PAR-1 in a dose-dependent manner (Fig. 8E and F), but with different Kd. In particular, NHL-a101 and F1 peptide were able to strongly bind PAR1 with Kd of 39 and 46 nmol/l, respectively, whereas NHL-a102 bound to PAR-1 with a lower affinity (Kd = 1861 nmol/l).

Finally, the vp17/PAR1 interaction was confirmed by performing a co-immunoprecipitation assay on Raji cells. Cells (1.5 × 10^6^) were incubated for 15 min with refp17 or a representative clonogenic vp17 (NHL-a102) (1.5 μg). Then, a chemical cross-linker was added to the cells and the formation of a covalent complex was analyzed by western blot analysis. A band of ~95 kDa, corresponding to dimeric vp17 NHL-a102 (34 kDa) and PAR1 (60 kDa), was detected in Raji cells treated with NHL-a102, but not in not treated cells or treated with refp17 (Fig. 8G). Moreover, a band of 60 KDa corresponding to PAR1 was detected in Raji cell lysate.

Overall, these data confirm the ability of vp17s, but not refp17, to interact with PAR1 to induce B-cell clonogenicity.

## Discussion

In the present study, we explored the molecular mechanisms through which vp17s promote B-cell proliferation and demonstrated that the clonogenic activity of these variants is triggered by PAR1-mediated EGFR transactivation through G proteins of the Gq family and a MMPs-dependent process. PAR1 is known to promote tumor progression [33] and belong to the large family of GPCRs, which represent an ideal target for lymphoma therapeutics [34]. Moreover, PAR1 ligand represents a survival factor for malignant cells and a clinically relevant cellular resistance factor [35]. Therefore, our data suggest that PAR1 might represent an attractive drug target and a novel tool to counteract the B-cell clonogenic potential of vp17s, thus potentially improving the control of HIV-1-related lymphomas. Proteolytic activation of PARs requires unmasking of a tethered ligand, which binds intramolecularly to the body of the receptor stabilizing an active conformation. This triggers a transmembrane signaling and the consequent interaction with the subunits of heterotrimeric G proteins [36]. Surprisingly, both NHL-a101 and NHL-a102 as well as the F1 peptide (aa 2–21 of the viral protein) do not show any significant sequence homology with the known PAR1 ligand sequence. However, several recent studies suggested that different agonists or synthetic peptide ligands can elicit distinct signaling responses through the activation of the same PAR [37]. This process, named “functional selectivity” or “biased agonism” has been reported for many GPCRs and involves ligand-induced stabilization of distinct active receptor conformations, which may be facilitated by receptor compartmentalization within plasma membrane microdomains [38]. In the case of receptor homo- or heterodimers, such as PAR1-PAR1 or PAR1-PAR4 dimers, the ability of selected ligands to drive different receptor conformations is even more complex than in the case of receptor monomers [33, 39]. Therefore, future studies involving molecular modeling and dynamics will be useful to elucidate the structural and functional mechanisms through which the clonogenic vp17s, unlike refp17, can activate PAR1 signaling.

Previous studies indicated that EGFR may contribute to the development of haematological malignancies and suggested a possible inhibition of EGFR-mediated signaling for therapeutic purposes [24, 40, 41]. Our findings identify EGFR transactivation as an essential link between PAR1 activation and B cell proliferation. Numerous investigations have revealed that GPCRs, like PARs, are able to exploit EGFR as a downstream signaling partner for generating potent mitogenic signals contributing to cancer development and progression [42, 43]. In particular, EGFR transactivation by GPCRs has been linked to the modulation of cell proliferation in a variety of cancer types and other diseases, becoming a paradigm for inter-receptor cross-talk [30, 33]. Moreover, EGFR overexpression was associated with drug-resistance of diffuse large B-cell lymphoma (DLBCL) [41], the most common subtype of NHL. Due to the role of EGFR in promoting various cancers, including lung and oral cancer [33, 44], we can hypothesize that the mechanism used by vp17s, which involves the PAR1-triggered EGFR transactivation, might be also extended, in addition to aggressive B-cell NHLs, also to other AIDS-defining cancers, like Kaposi’s sarcoma and cervical cancer or to non-AIDS-defining cancers, as Hodgkin lymphoma or cancer of the oral cavity/pharynx, lung, anus and liver [45, 46]. It is worth noting that clonogenic vp17s, but not refp17, are able to promote triple-negative breast cancer cell proliferation in vitro [47]. Thus, therapeutic agents, as tyrosine kinase inhibitors (erlotinib and gefinitib approved for the treatment of patients with non-small-cell lung cancer) or monoclonal antibodies (cetuximab for metastatic colorectal cancer, head and neck), which bind the tyrosine kinase and extracellular domain of EGFR, respectively, blocking its transactivation, might be considered as potential inhibitors of vp17-mediated B-cell clonogenicity in the setting of HIV lymphomagenesis.

Our data also point to a possible pathogenic role of HER2, which is known to form heterodimers with EGFR to transduce mitogenic signals, in mediating the growth-promoting activity of vp17s. These findings could suggest the possible use in the HIV setting of the known drugs, which target HER2 and downstream proliferative signaling pathways, as trastuzumab, pertuzumab, trastuzumab emtansine (T-DM1) and lapatinib, used clinically in treating HER2^+^ breast cancer [48–50].

Notably, our study, employing high-throughput antibody array technology and immunoblot analysis has provided important and novel insights into the signaling network triggered by clonogenic vp17s. These variant matrix proteins, in contrast to refp17, trigger the EGFR/PI3K/Akt signaling cascade, which plays a central and crucial role in modulating different molecules involved in cell cycle and cancer progression. Through an analysis encompassing literature mining, data enrichment via Gene Ontology and protein–protein interaction database STRING, we built a signal transduction pathway (Fig. 1) highlighting how Akt activation can concur to modulate the activity of a variety of molecules involved in cell cycle progression and proliferation, as CDK1 [51] and consequently Rb [52], p53 [53]—through NPM binding [54, 55]—, PTP-1B [56, 57] and STAT1 [58]. Modulation of PTP-1B and STAT1 is known to be directly strengthened by EGFR activation [56, 59]. Moreover, PI3K is also able to activate ABL1 [60], p53—through MAPK8 binding [61]—and RAC1 [62], which regulate transcription in the G, G1/S and G2/M phase transition, respectively (Fig. 1). In addition, when we compared the signaling molecules triggered by both vp17s, we observed that NHL-a101, but not NHL-a102, modulated the function of CHEK1—through the PI3K/Akt axis [63]—, CHEK2—through a complex with p53 and hMSH2 related to the DNA damage [64, 65]—, CDK4 [66], and JAK-1 [67], all contributing to cell survival and proliferation. On the other side, NHL-a102, differently from NHL-a101, modulated MAPK8 and CDK2 through PI3K/AKT activation, thus concurring in turn to modulate the function of p53 [61] and Rb [68], respectively, two regulators of the main tumor-suppressor pathways. Indeed, the blockade of EGFR tyrosine kinase activity by AG1478 and blockade of PI3K/Akt signaling pathway by specific inhibitors, as WT and Akt inhibitor VIII, resulted in robust inhibition of EGFR-induced B-cell proliferation. The triggering of EGFR/PI3K/Akt signaling cascade has been previously observed in response to PAR1 stimulation [29, 33] and this is in line with several observations supporting a central role of this pathway in mediating the malignant progression of several human cancers [25].

Our data also show that the B-cell clonogenic activity of vp17s is mediated by MMPs, which are shedding agents able to generate soluble and bioactive ligands for EGFR activation [28]. In particular, inhibition of MMPs with two different broad-spectrum inhibitors, like Batimastat and Ilomastat, significantly suppressed vp17s-triggered B-cell colony formation. PAR1 is known to enhance cell proliferation through an MMP-dependent EGFR transactivation [29] and elevated levels of MMPs have been correlated with a poor clinical outcome of patients with NHL [69]. Moreover, there is an emergent evidence of the MMP role in cancer formation and progression, and there is a growing interest to exploit MMPs as therapeutic targets [70]. Therefore, MMPs might represent, in HIV^+^ patients, additional attractive therapeutic targets, which might contribute to improve the management of cancer in this setting.

Finally, since our data show that the clonogenic activity of the F1 peptide is driven by the PAR1/EGFR/PI3K/Akt signaling pathway, as for vp17s. Therefore, we can hypothesize that the AT20-based therapeutic vaccine for HIV-1 (AT20 sequence: aa 9–28 of matrix protein), able to induce an anti-p17 neutralizing antibody response and successfully passed through phase I clinical trial [71, 72], might be of potential preventive relevance to avoid or delay the insurgence of malignant lymphomas in HIV^+^ patients.

Taken together, the data presented here identify the mechanism through which HIV-1 vp17s can promote B-cell proliferation, suggesting that, from a therapeutic point of view, new strategies that exploit drugs targeting PAR1, EGFR, HER2, and MMPs, might prove useful to inhibit the growth-promoting and clonogenic activity of vp17s and therefore contributing to a more effective treatment of HIV-related lymphomas and other cancers associated with HIV- infection.

## References

[1] Rubinstein PG, Aboulafia DM, Zloza A (2014). Malignancies in HIV/AIDS: from epidemiology to therapeutic challenges. AIDS..

[2] Rabkin C (1994). Epidemiology of AIDS‐related malignancies. Curr Opin Oncol..

[3] He B, Qiao X, Klasse PJ, Chiu A, Chadburn A, Knowles DM (2006). HIV-1 envelope triggers polyclonal Ig class switch recombination through a CD40-independent mechanism involving BAFF and C-type lectin receptors. J Immunol.

[4] Kundu RK, Sangiorgi F, Wu LY, Pattengale PK, Hinton DR, Gill PS (1999). Expression of the human immunodeficiency virus-Tat gene in lymphoid tissues of transgenic mice is associated with B-cell lymphoma. Blood..

[5] Popovic M, Tenner-Racz K, Pelser C, Stellbrink H-J, van Lunzen J, Lewis G (2005). Persistence of HIV-1 structural proteins and glycoproteins in lymph nodes of patients under highly active antiretroviral therapy. Proc Natl Acad Sci USA..

[6] Caccuri F, Marsico S, Fiorentini S, Caruso A, Giagulli C (2016). HIV-1 Matrix Protein p17 and its Receptors. Curr Drug Targets..

[7] Vandergeeten C, Quivy V, Moutschen M, Van Lint C, Piette J, Legrand-Poels S (2007). HIV-1 protease inhibitors do not interfere with provirus transcription and host cell apoptosis induced by combined treatment TNF-alpha+TSA. Biochem Pharmacol..

[8] Caccuri F, Giagulli C, Bugatti A, Benetti A, Alessandri G, Ribatti D (2012). HIV-1 matrix protein p17 promotes angiogenesis via chemokine receptors CXCR1 and CXCR2. Proc Natl Acad Sci USA.

[9] Caccuri F, Rueckert C, Giagulli C, Schulze K, Basta D, Zicari S (2014). HIV-1 matrix protein p17 promotes lymphangiogenesis and activates the endothelin-1/endothelin B receptor axis. Arterioscler Thromb Vasc Biol..

[10] Ma SP, Lin M, Liu HN, Yu JX (2012). Lymphangiogenesis in non-Hodgkin’s lymphoma and its correlation with cyclooxygenase-2 and vascular endothelial growth factor-C. Oncol Lett.

[11] Ribatti D, Nico B, Ranieri G, Specchia G, Vacca A (2013). The role of angiogenesis in human non-Hodgkin lymphomas. Neoplasia..

[12] Dolcetti R, Giagulli C, He W, Selleri M, Caccuri F, Eyzaguirre L (2015). Role of HIV-1 matrix protein p17 variants in lymphoma pathogenesis. Proc Natl Acad Sci USA..

[13] Caccuri F, Muraro E, Gloghini A, Turriziani O, Riminucci M, Giagulli C (2019). Lymphomagenic properties of a HIV p17 variant derived from a splenic marginal zone lymphoma occurred in a HIV-infected patient. Hematol Oncol.

[14] He W, Mazzuca P, Yuan W, Varney K, Bugatti A, Cagnotto A (2019). Identification of amino acid residues critical for the B cell growth-promoting activity of HIV-1 matrix protein p17 variants. Biochim Biophys Acta Gen Subj..

[15] Giagulli C, Marsico S, Magiera AK, Bruno R, Caccuri F, Barone I (2011). Opposite effects of HIV-1 p17 variants on PTEN activation and cell growth in B cells. PLoS ONE..

[16] Giagulli C, D’Ursi P, He W, Zorzan S, Caccuri F, Varney K (2017). A single amino acid substitution confers B-cell clonogenic activity to the HIV-1 matrix protein p17. Sci Rep..

[17] Cheadle C, Vawter MP, Freed WJ, Becker KG (2003). Analysis of microarray data using Z score transformation. J Mol Diagn..

[18] von Mering C, Huynen M, Jaeggi D, Schmidt S, Bork P, Snel B (2003). STRING: a database of predicted functional associations between proteins. Nucleic Acids Res..

[19] Giagulli C, Caccuri F, Cignarella F, Lougaris V, Martorelli D, Bugatti A (2014). A CXCR1 haplotype hampers HIV-1 matrix protein p17 biological activity. AIDS..

[20] Khalifa MB, Choulier L, Lortat-Jacob H, Altschuh D, Vernet T (2001). BIACORE data processing: an evaluation of the global fitting procedure. Anal Biochem..

[21] Zeinolabediny Y, Caccuri F, Colombo L, Morelli F, Romeo M, Rossi A (2017). HIV-1 matrix protein p17 misfolding forms toxic amyloidogenic assemblies that induce neurocognitive disorders. Sci Rep..

[22] Pawson T, Nash P (2000). Protein-protein interactions define specificity in signal transduction. Genes Dev..

[23] Mattoon DR, Lamothe B, Lax I, Schlessinger J (2004). The docking protein Gab1 is the primary mediator of EGF-stimulated activation of the PI-3K/Akt cell survival pathway. BMC Biol..

[24] Saryeddine L, Zibara K, Kassem N, Badran B, El-Zein N (2016). EGF-Induced VEGF Exerts a PI3K-Dependent Positive Feedback on ERK and AKT through VEGFR2 in Hematological In Vitro Models. PLoS ONE..

[25] Roskoski R (2014). The ErbB/HER family of protein-tyrosine kinases and cancer. Pharm Res..

[26] Bhola NE, Grandis JR (2008). Crosstalk between G-protein-coupled receptors and epidermal growth factor receptor in cancer. Front Biosci..

[27] Uchiyama-Tanaka Y, Matsubara H, Mori Y, Kosaki A, Kishimoto N, Amano K (2002). Involvement of HB-EGF and EGF receptor transactivation in TGF-β-mediated fibronectin expression in mesangial cells. Kidney Int..

[28] Prenzel N, Zwick E, Daub H, Leserer M, Abraham R, Wallasch C (1999). EGF receptor transactivation by G-protein-coupled receptors requires metalloproteinase cleavage of proHB-EGF. Nature..

[29] Huang C-Y, Lin H-J, Chen H-S, Cheng S-Y, Hsu H-C, Tang C-H (2013). Thrombin promotes matrix metalloproteinase-13 expression through the PKCδ/c-Src/EGFR/PI3K/Akt/AP-1 signaling pathway in human chondrocytes. Mediat Inflamm..

[30] Gschwind A, Zwick E, Prenzel N, Leserer M, Ullrich A (2001). Cell communication networks: epidermal growth factor receptor transactivation as the paradigm for interreceptor signal transmission. Oncogene..

[31] Wang Z (2016). Transactivation of epidermal growth factor receptor by G protein coupled receptors: recent progress, challenges and future research. Int J Mol Sci..

[32] Han N, Jin K, He K, Cao J, Teng L (2011). Protease-activated receptors in cancer: a systematic review. Oncol Lett.

[33] Gieseler F, Ungefroren H, Settmacher U, Hollenberg MD, Kaufmann R (2013). Proteinase-activated receptors (PARs) – focus on receptor-receptor-interactions and their physiological and pathophysiological impact. Cell Commun Signal..

[34] Nugent A, Proia RL (2017). The role of G protein-coupled receptors in lymphoid malignancies. Cell Signal.

[35] Schiller H, Bartscht T, Arlt A, Zahn MO, Seifert A, Bruhn T (2002). Thrombin as a survival factor for cancer cells: thrombin activation in malignant effusions in vivo and inhibition of idarubicin-induced cell death in vitro. Int J Clin Pharm Ther..

[36] Oldham WM, Hamm HE (2007). How do receptors activate G proteins?. Adv Protein Chem.

[37] Russo A, Soh UJ, Trejo J (2009). Proteases display biased agonism at protease-activated receptors: location matters!. Mol Interv..

[38] Urban JD, Clarke WP, von Zastrow M, Nichols DE, Kobilka B, Weinstein H (2007). Functional selectivity and classical concepts of quantitative pharmacology. J Pharm Exp Ther..

[39] Kenakin T (2013). New concepts in pharmacological efficacy at 7TM receptors: IUPHAR review 2. Br J Pharmacol.

[40] Sun JZ, Lu Y, Xu Y, Liu F, Li F-Q, Wang Q-L (2012). Epidermal growth factor receptor expression in acute myelogenous leukaemia is associated with clinical prognosis. Hematol Oncol..

[41] Jin J, Wang L, Tao Z, Zhang J, Lv F, Cao J (2020). PDGFD induces ibrutinib resistance of diffuse large B-cell lymphoma through activation of EGFR. Mol Med Rep..

[42] Daub H, Weiss FU, Wallasch C, Ullrich A (1996). Role of transactivation of the EGF receptor in signalling by G-protein-coupled receptors. Nature..

[43] New DC, Wong YH (2007). Molecular mechanisms mediating the G protein-coupled receptor regulation of cell cycle progression. J Mol Signal..

[44] Brusevold I, Tveteraas IH, Aasrum M, Ødegård J, Sandnes DL, Christoffersen T (2014). Role of LPAR3, PKC and EGFR in LPA-induced cell migration in oral squamous carcinoma cells. BMC Cancer..

[45] Wang CC, Silverberg MJ, Abrams DI (2014). Non-AIDS-defining malignancies in the HIV-infected population. Curr Infect Dis Rep..

[46] Hernández-Ramírez RU, Shiels MS, Dubrow R, Engels EA (2017). Cancer risk in HIV-infected people in the USA from 1996 to 2012: a population-based, registry-linkage study. Lancet Hiv..

[47] Caccuri F, Giordano F, Barone I, Mazzucca P, Giagulli C, Andò S (2017). S. HIV-1 matrix protein p17 and its variants promote human triple negative breast cancer cell aggressiveness. Infect Agent Cancer.

[48] Verma S, Miles D, Gianni L, Krop IE, Welslau M, Baselga J (2012). Trastuzumab emtansine for HER2-positive advanced breast cancer. N. Engl J Med..

[49] Rimawi MF, Mayer IA, Forero A, Nanda R, Goetz MP, Rodriguez AA (2013). Multicenter phase II study of neoadjuvant lapatinib and trastuzumab with hormonal therapy and without chemotherapy in patients with human epidermal growth factor receptor 2-overexpressing breast cancer: TBCRC 006. J Clin Oncol..

[50] Robidoux A, Tang G, Rastogi P, Geyer CE, Azar CA, Atkins JN (2013). Lapatinib as a component of neoadjuvant therapy for HER2-positive operable breast cancer (NSABP protocol B-41): An open-label, randomised phase 3 trial. Lancet Oncol.

[51] Shtivelman E, Sussman J, Stokoe D (2002). A role for PI 3-kinase and PKB activity in the G2/M phase of the cell cycle. Curr Biol.

[52] Liu T, Zhu E, Wang L, Okada T, Yamaguchi A, Okada N (2005). Abnormal expression of Rb pathway-related proteins in salivary gland acinic cell carcinoma. Hum Pathol..

[53] Colombo E, Marine JC, Danovi D, Falini B, Pelicci PG (2002). Nucleophosmin regulates the stability and transcriptional activity of p53. Nat Cell Biol..

[54] Lee SB, Xuan Nguyen TL, Choi JW, Lee K-H, Cho S-W, Liu Z (2008). Nuclear Akt interacts with B23/NPM and protects it from proteolytic cleavage, enhancing cell survival. Proc Natl Acad Sci USA.

[55] Koike A, Nishikawa H, Wu W, Okada Y, Venkitaraman AR, Ohta T (2010). Recruitment of phosphorylated NPM1 to sites of DNA damage through RNF8-dependent ubiquitin conjugates. Cancer Res..

[56] Ravichandran LV, Chen H, Li Y, Quon MJ (2001). Phosphorylation of PTP1B at Ser(50) by Akt impairs its ability to dephosphorylate the insulin receptor. Mol Endocrinol..

[57] Dube N, Tremblay ML (2005). Involvement of the small protein tyrosine phosphatases TC-PTP and PTP1B in signal transduction and diseases: from diabetes, obesity to cell cycle, and cancer. Biochim Biophys Acta..

[58] Frank DA, Mahajan S, Ritz J (1997). B lymphocytes from patients with chronic lymphocytic leukemia contain signal transducer and activator of transcription (STAT) 1 and STAT3 constitutively phosphorylated on serine residues. J Clin Invest..

[59] Schmitt NC, Trivedi S, Ferris RL (2015). STAT1 Activation is enhanced by cisplatin and variably affected by EGFR inhibition in HNSCC Cells. Mol Cancer Ther..

[60] Sattler M, Salgia R, Okuda K, Uemura N, Durstin MA, Pisick E (1996). The proto-oncogene product p120CBL and the adaptor proteins CRKL and c-CRK link c-ABL, p190BCR/ABL and p210BCR/ABL to the phosphatidylinositol-3’ kinase pathway. Oncogene.

[61] Xue Y, Ramaswamy NT, Hong X, Pelling JC (2003). Association of JNK1 with p21waf1 and p53: modulation of JNK1 activity. Mol Carcinog..

[62] Wu CY, Carpenter ES, Takeuchi KK, Halbrook CJ, Peverley LV, Bien H (2014). PI3K regulation of RAC1 is required for KRAS-induced pancreatic tumorigenesis in mice. Gastroenterology.

[63] Xu N, Lao Y, Zhang Y, Gillespie DA (2012). Akt: a double-edged sword in cell proliferation and genome stability. J Oncol..

[64] Luo Y, Lin FT, Lin WC (2004). ATM-mediated stabilization of hMutL DNA mismatch repair proteins augments p53 activation during DNA damage. Mol Cell Biol.

[65] Aliouat-Denis CM, Dendouga N, Van den Wyngaert I, Goehlmann H, Steller U, van de Weyer I (2005). p53-independent regulation of p21Waf1/Cip1 expression and senescence by Chk2. Mol Cancer Res..

[66] Tang LH, Contractor T, Clausen R, Klimstra DS, Nancy Du Y-C, Allen PJ (2012). Attenuation of the retinoblastoma pathway in pancreatic neuroendocrine tumors due to increased cdk4/cdk6. Clin Cancer Res..

[67] Danial NN, Losman JA, Lu T, Yip N, Krisnan K, Krolewski J (1998). Direct interaction of Jak1 and v-Abl is required for v-Abl-induced activation of STATs and proliferation. Mol Cell Biol.

[68] Li W, Kotoshiba S, Berthet C, Hilton MB, Kaldis P (2009). Rb/Cdk2/Cdk4 triple mutant mice elicit an alternative mechanism for regulation of the G1/S transition. Proc Natl Acad Sci USA.

[69] Kossakowska AE, Urbanski SJ, Janowska-Wieczorek A (2000). Matrix metalloproteinases and their tissue inhibitors - expression, role and regulation in human malignant non-Hodgkin’s lymphomas. Leuk Lymphoma..

[70] Duffy MJ, Mullooly M, O’Donovan N, Sukor S, Crown J, Pierce A (2011). The ADAMs family of proteases: new biomarkers and therapeutic targets for cancer?. Clin Proteom..

[71] Fiorentini S, Marsico S, Becker PD, Iaria ML, Bruno R, Guzmàn CA (2008). Synthetic peptide AT20 coupled to KLH elicits antibodies against a conserved conformational epitope from a major functional area of the HIV-1 matrix protein p17. Vaccine..

[72] Iaria ML, Fiorentini S, Focà E, Zicari S, Giagulli C, Caccuri F (2014). Synthetic HIV-1 matrix protein p17-based AT20-KLH therapeutic immunization in HIV-1-infected patients receiving antiretroviral treatment: A phase I safety and immunogenicity study. Vaccine..

